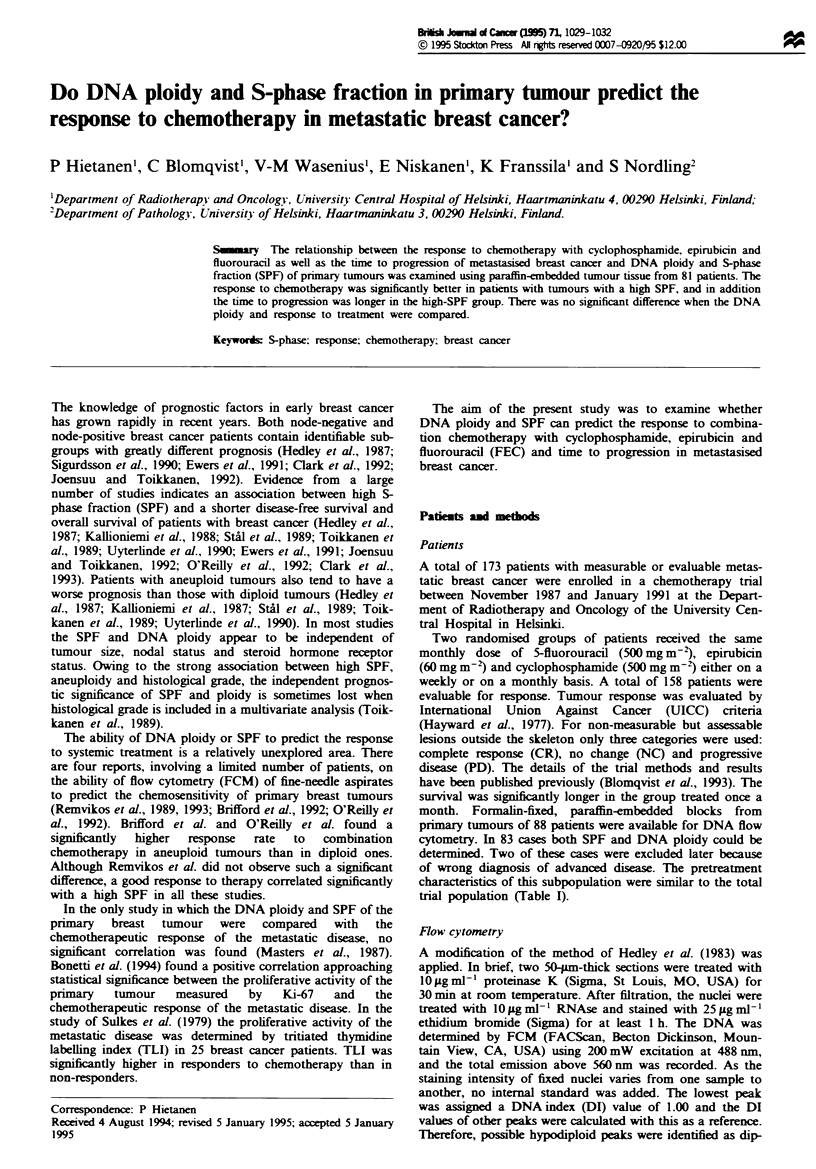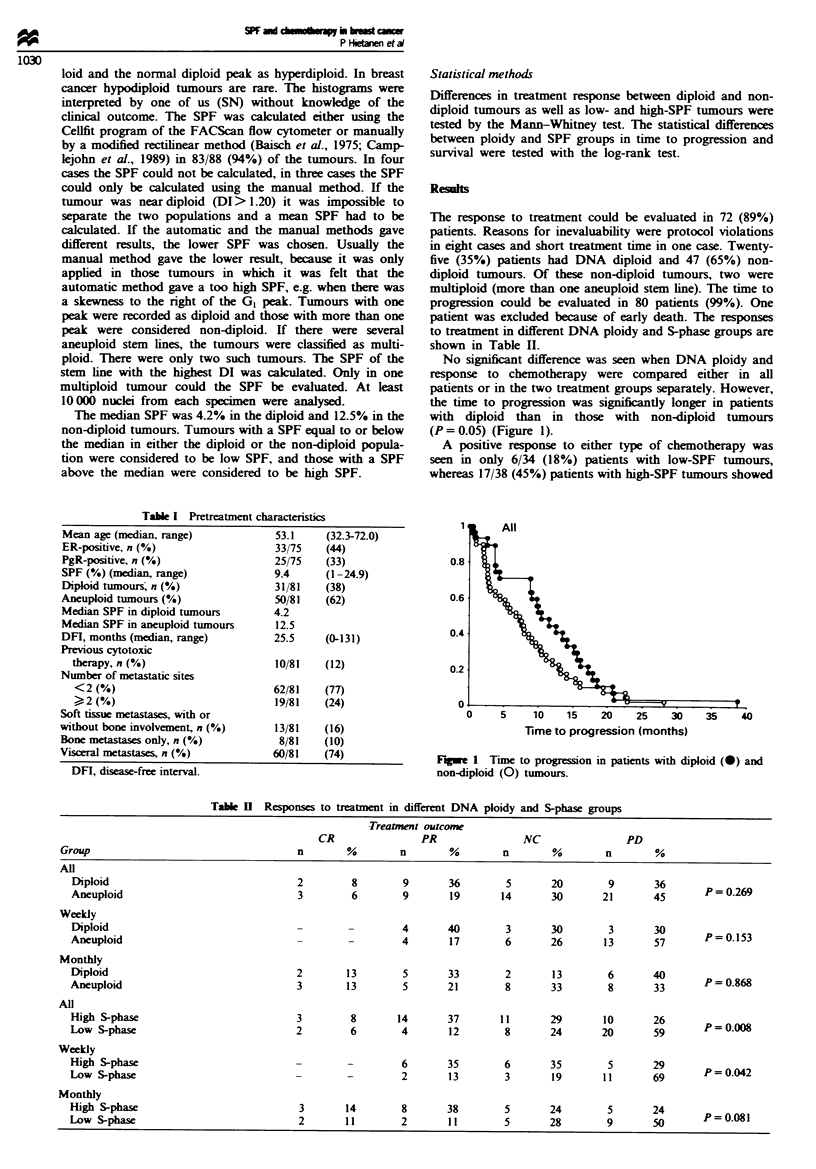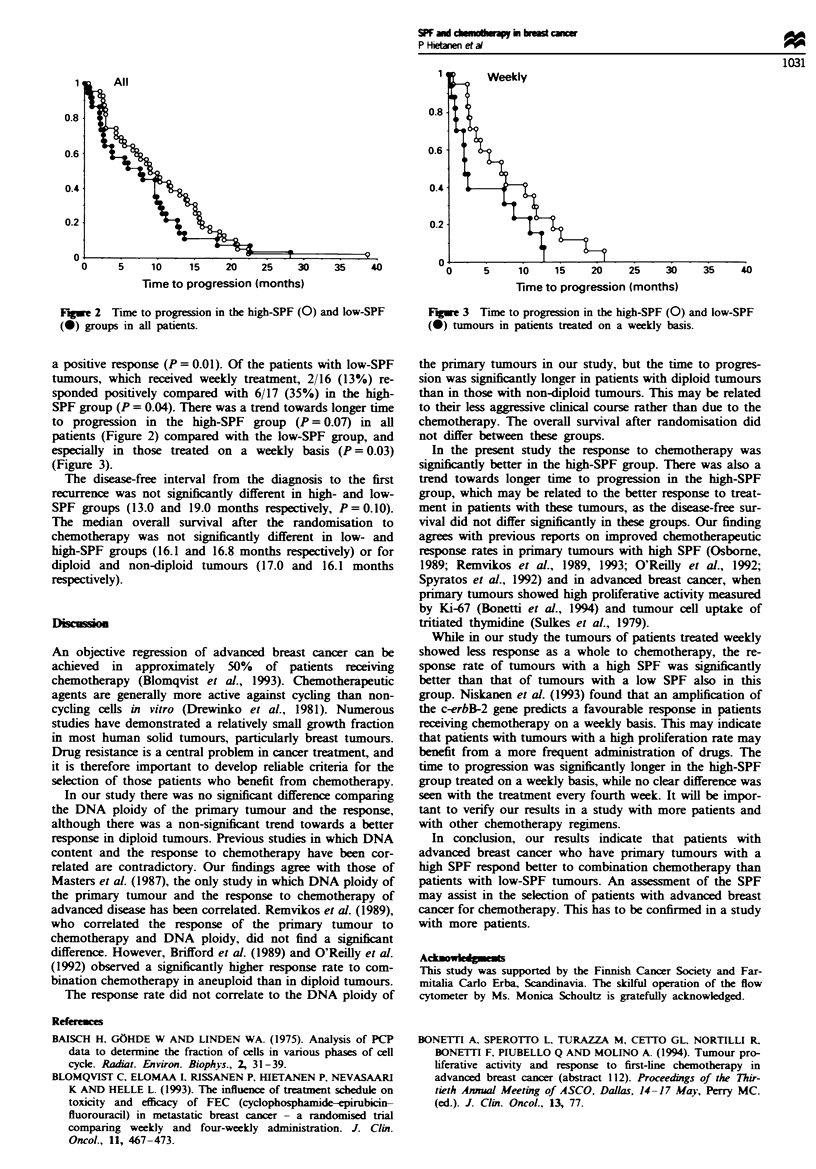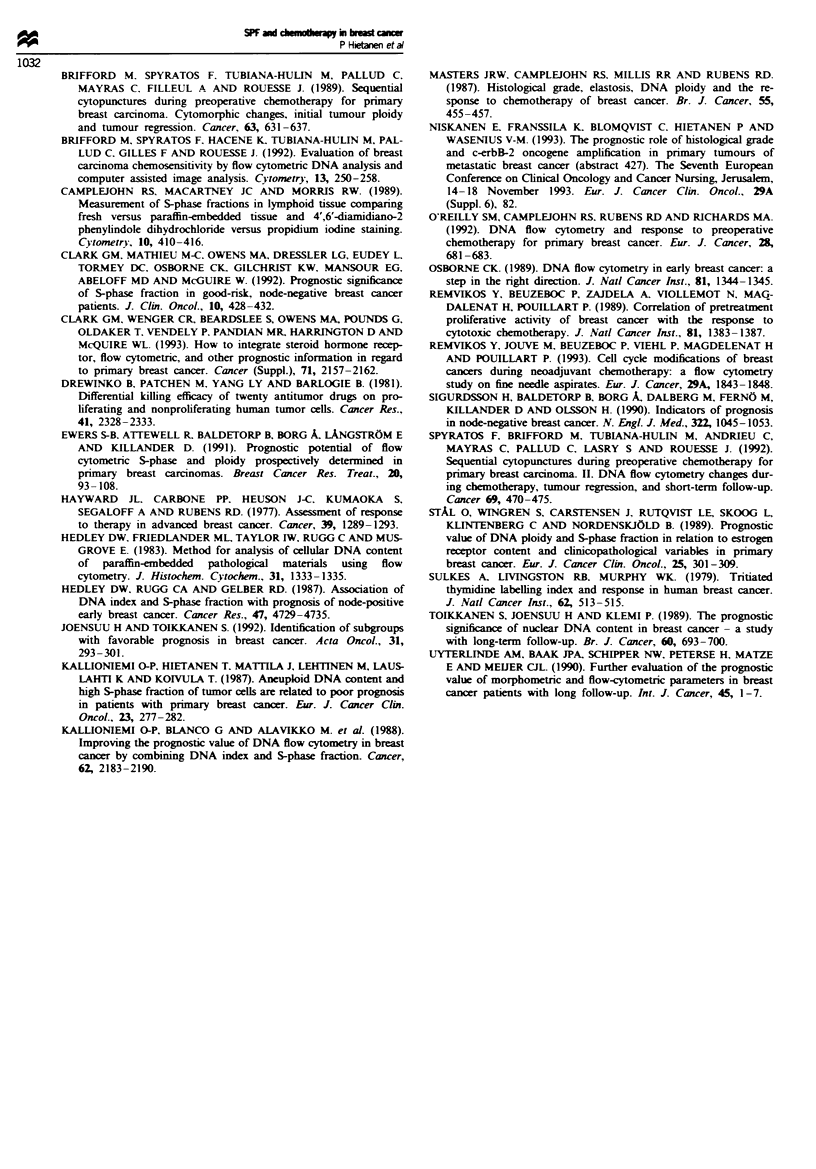# Do DNA ploidy and S-phase fraction in primary tumour predict the response to chemotherapy in metastatic breast cancer?

**DOI:** 10.1038/bjc.1995.198

**Published:** 1995-05

**Authors:** P. Hietanen, C. Blomqvist, V. M. Wasenius, E. Niskanen, K. Franssila, S. Nordling

**Affiliations:** Department of Radiotherapy and Oncology, University Central Hospital of Helsinki, Finland.

## Abstract

The relationship between the response to chemotherapy with cyclophosphamide, epirubicin and fluorouracil as well as the time to progression of metastasised breast cancer and DNA ploidy and S-phase fraction (SPF) of primary tumours was examined using paraffin-embedded tumour tissue from 81 patients. The response to chemotherapy was significantly better in patients with tumours with a high SPF, and in addition the time to progression was longer in the high-SPF group. There was no significant difference when the DNA ploidy and response to treatment were compared.


British Journal of Cancer (1995) 71, 1029-1032

© 1995 Stockton Press All rights reserved 0007-0920/95 $12.00

### Do DNA ploidy and S-phase fraction in primary tumour predict the response to chemotherapy in metastatic breast cancer?

P Hietanen[1], C Blomqvist[1], V-M Wasenius[1], E Niskanen[1], K Franssila[1] and S Nordling[2]

[1]*Department of Radiotherapy and Oncology, University Central Hospital of Helsinki, Haartmaninkatu 4, 00290 Helsinki, Finland;* [2]*Department of Pathology, University of Helsinki, Haartmaninkatu 3, 00290 Helsinki, Finland.*

**Summary** The relationship between the response to chemotherapy with cyclophosphamide, epirubicin and fluorouracil as well as the time to progression of metastasised breast cancer and DNA ploidy and S-phase fraction (SPF) of primary tumours was examined using paraffin-embedded tumour tissue from 81 patients. The response to chemotherapy was significantly better in patients with tumours with a high SPF, and in addition the time to progression was longer in the high-SPF group. There was no significant difference when the DNA ploidy and response to treatment were compared.

**Keywords:** S-phase; response; chemotherapy; breast cancer

The knowledge of prognostic factors in early breast cancer has grown rapidly in recent years. Both node-negative and node-positive breast cancer patients contain identifiable subgroups with greatly different prognosis (Hedley *et al.*, 1987; Sigurdsson *et al.*, 1990; Ewers *et al.*, 1991; Clark *et al.*, 1992; Joensuu and Toikkanen, 1992). Evidence from a large number of studies indicates an association between high S-phase fraction (SPF) and a shorter disease-free survival and overall survival of patients with breast cancer (Hedley *et al.*, 1987; Kallioniemi *et al.*, 1988; Stål *et al.*, 1989; Toikkanen *et al.*, 1989; Uyterlinde *et al.*, 1990; Ewers *et al.*, 1991; Joensuu and Toikkanen, 1992; O'Reilly *et al.*, 1992; Clark *et al.*, 1993). Patients with aneuploid tumours also tend to have a worse prognosis than those with diploid tumours (Hedley *et al.*, 1987; Kallioniemi *et al.*, 1987; Stål *et al.*, 1989; Toikkanen *et al.*, 1989; Uyterlinde *et al.*, 1990). In most studies the SPF and DNA ploidy appear to be independent of tumour size, nodal status and steroid hormone receptor status. Owing to the strong association between high SPF, aneuploidy and histological grade, the independent prognostic significance of SPF and ploidy is sometimes lost when histological grade is included in a multivariate analysis (Toikkanen *et al.*, 1989).

The ability of DNA ploidy or SPF to predict the response to systemic treatment is a relatively unexplored area. There are four reports, involving a limited number of patients, on the ability of flow cytometry (FCM) of fine-needle aspirates to predict the chemosensitivity of primary breast tumours (Remvikos *et al.*, 1989, 1993; Brifford *et al.*, 1992; O'Reilly *et al.*, 1992). Brifford *et al.* and O'Reilly *et al.* found a significantly higher response rate to combination chemotherapy in aneuploid tumours than in diploid ones. Although Remvikos *et al.* did not observe such a significant difference, a good response to therapy correlated significantly with a high SPF in all these studies.

In the only study in which the DNA ploidy and SPF of the primary breast tumour were compared with the chemotherapeutic response of the metastatic disease, no significant correlation was found (Masters *et al.*, 1987). Bonetti *et al.* (1994) found a positive correlation approaching statistical significance between the proliferative activity of the primary tumour measured by Ki-67 and the chemotherapeutic response of the metastatic disease. In the study of Sulkes *et al.* (1979) the proliferative activity of the metastatic disease was determined by tritiated thymidine labelling index (TLI) in 25 breast cancer patients. TLI was significantly higher in responders to chemotherapy than in non-responders.

Correspondence: P Hietanen
Received 4 August 1994; revised 5 January 1995; accepted 5 January 1995

The aim of the present study was to examine whether DNA ploidy and SPF can predict the response to combination chemotherapy with cyclophosphamide, epirubicin and fluorouracil (FEC) and time to progression in metastasised breast cancer.

### Patients and methods

### Patients

A total of 173 patients with measurable or evaluable metastatic breast cancer were enrolled in a chemotherapy trial between November 1987 and January 1991 at the Department of Radiotherapy and Oncology of the University Central Hospital in Helsinki.

Two randomised groups of patients received the same monthly dose of 5-fluorouracil (500 mg $m^{-2}$), epirubicin (60 mg $m^{-2}$) and cyclophosphamide (500 mg $m^{-2}$) either on a weekly or on a monthly basis. A total of 158 patients were evaluable for response. Tumour response was evaluated by International Union Against Cancer (UICC) criteria (Hayward *et al.*, 1977). For non-measurable but assessable lesions outside the skeleton only three categories were used: complete response (CR), no change (NC) and progressive disease (PD). The details of the trial methods and results have been published previously (Blomqvist *et al.*, 1993). The survival was significantly longer in the group treated once a month. Formalin-fixed, paraffin-embedded blocks from primary tumours of 88 patients were available for DNA flow cytometry. In 83 cases both SPF and DNA ploidy could be determined. Two of these cases were excluded later because of wrong diagnosis of advanced disease. The pretreatment characteristics of this subpopulation were similar to the total trial population (Table I).

### Flow cytometry

A modification of the method of Hedley *et al.* (1983) was applied. In brief, two 50-μm-thick sections were treated with 10 μg $ml^{-1}$ proteinase K (Sigma, St Louis, MO, USA) for 30 min at room temperature. After filtration, the nuclei were treated with 10 μg $ml^{-1}$ RNAse and stained with 25 μg $ml^{-1}$ ethidium bromide (Sigma) for at least 1 h. The DNA was determined by FCM (FACScan, Becton Dickinson, Mountain View, CA, USA) using 200 mW excitation at 488 nm, and the total emission above 560 nm was recorded. As the staining intensity of fixed nuclei varies from one sample to another, no internal standard was added. The lowest peak was assigned a DNA index (DI) value of 1.00 and the DI values of other peaks were calculated with this as a reference. Therefore, possible hypodiploid peaks were identified as diploid and the normal diploid peak as hyperdiploid. In breast cancer hypodiploid tumours are rare. The histograms were interpreted by one of us (SN) without knowledge of the clinical outcome. The SPF was calculated either using the Cellfit program of the FACScan flow cytometer or manually by a modified rectilinear method (Baisch *et al.*, 1975; Camplejohn *et al.*, 1989) in 83/88 (94%) of the tumours. In four cases the SPF could not be calculated, in three cases the SPF could only be calculated using the manual method. If the tumour was near diploid (DI > 1.20) it was impossible to separate the two populations and a mean SPF had to be calculated. If the automatic and the manual methods gave different results, the lower SPF was chosen. Usually the manual method gave the lower result, because it was only applied in those tumours in which it was felt that the automatic method gave a too high SPF, e.g. when there was a skewness to the right of the $G_1$ peak. Tumours with one peak were recorded as diploid and those with more than one peak were considered non-diploid. If there were several aneuploid stem lines, the tumours were classified as multiploid. There were only two such tumours. The SPF of the stem line with the highest DI was calculated. Only in one multiploid tumour could the SPF be evaluated. At least 10 000 nuclei from each specimen were analysed.

The median SPF was 4.2% in the diploid and 12.5% in the non-diploid tumours. Tumours with a SPF equal to or below the median in either the diploid or the non-diploid population were considered to be low SPF, and those with a SPF above the median were considered to be high SPF.

*Statistical methods*

Differences in treatment response between diploid and non-diploid tumours as well as low- and high-SPF tumours were tested by the Mann–Whitney test. The statistical differences between ploidy and SPF groups in time to progression and survival were tested with the log-rank test.

### Results

The response to treatment could be evaluated in 72 (89%) patients. Reasons for inevaluability were protocol violations in eight cases and short treatment time in one case. Twenty-five (35%) patients had DNA diploid and 47 (65%) non-diploid tumours. Of these non-diploid tumours, two were multiploid (more than one aneuploid stem line). The time to progression could be evaluated in 80 patients (99%). One patient was excluded because of early death. The responses to treatment in different DNA ploidy and S-phase groups are shown in Table II.

No significant difference was seen when DNA ploidy and response to chemotherapy were compared either in all patients or in the two treatment groups separately. However, the time to progression was significantly longer in patients with diploid than in those with non-diploid tumours ($P = 0.05$) (Figure 1).

A positive response to either type of chemotherapy was seen in only 6/34 (18%) patients with low-SPF tumours, whereas 17/38 (45%) patients with high-SPF tumours showed

**Table I** Pretreatment characteristics

| | | |
|---|---|---|
| Mean age (median, range) | 53.1 | (32.3-72.0) |
| ER-positive, *n* (%) | 33/75 | (44) |
| PgR-positive, *n* (%) | 25/75 | (33) |
| SPF (%) (median, range) | 9.4 | (1-24.9) |
| Diploid tumours, *n* (%) | 31/81 | (38) |
| Aneuploid tumours (%) | 50/81 | (62) |
| Median SPF in diploid tumours | 4.2 | |
| Median SPF in aneuploid tumours | 12.5 | |
| DFI, months (median, range) | 25.5 | (0-131) |
| Previous cytotoxic therapy, *n* (%) | 10/81 | (12) |
| Number of metastatic sites | | |
| <2 (%) | 62/81 | (77) |
| ⩾2 (%) | 19/81 | (24) |
| Soft tissue metastases, with or without bone involvement, *n* (%) | 13/81 | (16) |
| Bone metastases only, *n* (%) | 8/81 | (10) |
| Visceral metastases, *n* (%) | 60/81 | (74) |

DFI, disease-free interval.

**Figure 1** Time to progression in patients with diploid (●) and non-diploid (○) tumours.

**Table II** Responses to treatment in different DNA ploidy and S-phase groups

| | Treatment outcome | | | | | | | | |
|---|---|---|---|---|---|---|---|---|---|
| | *CR* | | *PR* | | *NC* | | *PD* | | |
| *Group* | n | % | n | % | n | % | n | % | |
| All | | | | | | | | | |
| Diploid | 2 | 8 | 9 | 36 | 5 | 20 | 9 | 36 | $P = 0.269$ |
| Aneuploid | 3 | 6 | 9 | 19 | 14 | 30 | 21 | 45 | |
| Weekly | | | | | | | | | |
| Diploid | – | – | 4 | 40 | 3 | 30 | 3 | 30 | $P = 0.153$ |
| Aneuploid | – | – | 4 | 17 | 6 | 26 | 13 | 57 | |
| Monthly | | | | | | | | | |
| Diploid | 2 | 13 | 5 | 33 | 2 | 13 | 6 | 40 | $P = 0.868$ |
| Aneuploid | 3 | 13 | 5 | 21 | 8 | 33 | 8 | 33 | |
| All | | | | | | | | | |
| High S-phase | 3 | 8 | 14 | 37 | 11 | 29 | 10 | 26 | $P = 0.008$ |
| Low S-phase | 2 | 6 | 4 | 12 | 8 | 24 | 20 | 59 | |
| Weekly | | | | | | | | | |
| High S-phase | – | – | 6 | 35 | 6 | 35 | 5 | 29 | $P = 0.042$ |
| Low S-phase | – | – | 2 | 13 | 3 | 19 | 11 | 69 | |
| Monthly | | | | | | | | | |
| High S-phase | 3 | 14 | 8 | 38 | 5 | 24 | 5 | 24 | $P = 0.081$ |
| Low S-phase | 2 | 11 | 2 | 11 | 5 | 28 | 9 | 50 | |

**Figure 2** Time to progression in the high-SPF (○) and low-SPF (●) groups in all patients.

**Figure 3** Time to progression in the high-SPF (○) and low-SPF (●) tumours in patients treated on a weekly basis.

a positive response ($P = 0.01$). Of the patients with low-SPF tumours, which received weekly treatment, 2/16 (13%) responded positively compared with 6/17 (35%) in the high-SPF group ($P = 0.04$). There was a trend towards longer time to progression in the high-SPF group ($P = 0.07$) in all patients (Figure 2) compared with the low-SPF group, and especially in those treated on a weekly basis ($P = 0.03$) (Figure 3).

The disease-free interval from the diagnosis to the first recurrence was not significantly different in high- and low-SPF groups (13.0 and 19.0 months respectively, $P = 0.10$). The median overall survival after the randomisation to chemotherapy was not significantly different in low- and high-SPF groups (16.1 and 16.8 months respectively) or for diploid and non-diploid tumours (17.0 and 16.1 months respectively).

### Discussion

An objective regression of advanced breast cancer can be achieved in approximately 50% of patients receiving chemotherapy (Blomqvist *et al.*, 1993). Chemotherapeutic agents are generally more active against cycling than non-cycling cells *in vitro* (Drewinko *et al.*, 1981). Numerous studies have demonstrated a relatively small growth fraction in most human solid tumours, particularly breast tumours. Drug resistance is a central problem in cancer treatment, and it is therefore important to develop reliable criteria for the selection of those patients who benefit from chemotherapy.

In our study there was no significant difference comparing the DNA ploidy of the primary tumour and the response, although there was a non-significant trend towards a better response in diploid tumours. Previous studies in which DNA content and the response to chemotherapy have been correlated are contradictory. Our findings agree with those of Masters *et al.* (1987), the only study in which DNA ploidy of the primary tumour and the response to chemotherapy of advanced disease has been correlated. Remvikos *et al.* (1989), who correlated the response of the primary tumour to chemotherapy and DNA ploidy, did not find a significant difference. However, Brifford *et al.* (1989) and O'Reilly *et al.* (1992) observed a significantly higher response rate to combination chemotherapy in aneuploid than in diploid tumours.

The response rate did not correlate to the DNA ploidy of the primary tumours in our study, but the time to progression was significantly longer in patients with diploid tumours than in those with non-diploid tumours. This may be related to their less aggressive clinical course rather than due to the chemotherapy. The overall survival after randomisation did not differ between these groups.

In the present study the response to chemotherapy was significantly better in the high-SPF group. There was also a trend towards longer time to progression in the high-SPF group, which may be related to the better response to treatment in patients with these tumours, as the disease-free survival did not differ significantly in these groups. Our finding agrees with previous reports on improved chemotherapeutic response rates in primary tumours with high SPF (Osborne, 1989; Remvikos *et al.*, 1989, 1993; O'Reilly *et al.*, 1992; Spyratos *et al.*, 1992) and in advanced breast cancer, when primary tumours showed high proliferative activity measured by Ki-67 (Bonetti *et al.*, 1994) and tumour cell uptake of tritiated thymidine (Sulkes *et al.*, 1979).

While in our study the tumours of patients treated weekly showed less response as a whole to chemotherapy, the response rate of tumours with a high SPF was significantly better than that of tumours with a low SPF also in this group. Niskanen *et al.* (1993) found that an amplification of the c-*erb*B-2 gene predicts a favourable response in patients receiving chemotherapy on a weekly basis. This may indicate that patients with tumours with a high proliferation rate may benefit from a more frequent administration of drugs. The time to progression was significantly longer in the high-SPF group treated on a weekly basis, while no clear difference was seen with the treatment every fourth week. It will be important to verify our results in a study with more patients and with other chemotherapy regimens.

In conclusion, our results indicate that patients with advanced breast cancer who have primary tumours with a high SPF respond better to combination chemotherapy than patients with low-SPF tumours. An assessment of the SPF may assist in the selection of patients with advanced breast cancer for chemotherapy. This has to be confirmed in a study with more patients.

#### Acknowledgements
This study was supported by the Finnish Cancer Society and Farmitalia Carlo Erba, Scandinavia. The skilful operation of the flow cytometer by Ms. Monica Schoultz is gratefully acknowledged.

### References

BAISCH H, GÖHDE W AND LINDEN WA. (1975). Analysis of PCP data to determine the fraction of cells in various phases of cell cycle. *Radiat. Environ. Biophys.*, **2**, 31–39.

BLOMQVIST C, ELOMAA I, RISSANEN P, HIETANEN P, NEVASAARI K AND HELLE L. (1993). The influence of treatment schedule on toxicity and efficacy of FEC (cyclophosphamide–epirubicin–fluorouracil) in metastatic breast cancer – a randomised trial comparing weekly and four-weekly administration. *J. Clin. Oncol.*, **11**, 467–473.

BONETTI A, SPEROTTO L, TURAZZA M, CETTO GL, NORTILLI R, BONETTI F, PIUBELLO Q AND MOLINO A. (1994). Tumour proliferative activity and response to first-line chemotherapy in advanced breast cancer (abstract 112). *Proceedings of the Thirtieth Annual Meeting of ASCO, Dallas, 14–17 May*, Perry MC. (ed.). *J. Clin. Oncol.*, **13**, 77.

BRIFFORD M, SPYRATOS F, TUBIANA-HULIN M, PALLUD C, MAYRAS C, FILLEUL A AND ROUESSE J. (1989). Sequential cytopunctures during preoperative chemotherapy for primary breast carcinoma. Cytomorphic changes, initial tumour ploidy and tumour regression. *Cancer*, **63**, 631-637.

BRIFFORD M, SPYRATOS F, HACENE K, TUBIANA-HULIN M, PALLUD C, GILLES F AND ROUESSE J. (1992). Evaluation of breast carcinoma chemosensitivity by flow cytometric DNA analysis and computer assisted image analysis. *Cytometry*, **13**, 250-258.

CAMPLEJOHN RS, MACARTNEY JC AND MORRIS RW. (1989). Measurement of S-phase fractions in lymphoid tissue comparing fresh versus paraffin-embedded tissue and 4',6'-diamidiano-2 phenylindole dihydrochloride versus propidium iodine staining. *Cytometry*, **10**, 410-416.

CLARK GM, MATHIEU M-C, OWENS MA, DRESSLER LG, EUDEY L, TORMEY DC, OSBORNE CK, GILCHRIST KW, MANSOUR EG, ABELOFF MD AND McGUIRE W. (1992). Prognostic significance of S-phase fraction in good-risk, node-negative breast cancer patients. *J. Clin. Oncol.*, **10**, 428-432.

CLARK GM, WENGER CR, BEARDSLEE S, OWENS MA, POUNDS G, OLDAKER T, VENDELY P, PANDIAN MR, HARRINGTON D AND McQUIRE WL. (1993). How to integrate steroid hormone receptor, flow cytometric, and other prognostic information in regard to primary breast cancer. *Cancer* (Suppl.), **71**, 2157-2162.

DREWINKO B, PATCHEN M, YANG LY AND BARLOGIE B. (1981). Differential killing efficacy of twenty antitumor drugs on proliferating and nonproliferating human tumor cells. *Cancer Res.*, **41**, 2328-2333.

EWERS S-B, ATTEWELL R, BALDETORP B, BORG Å, LÅNGSTRÖM E AND KILLANDER D. (1991). Prognostic potential of flow cytometric S-phase and ploidy prospectively determined in primary breast carcinomas. *Breast Cancer Res. Treat.*, **20**, 93-108.

HAYWARD JL, CARBONE PP, HEUSON J-C, KUMAOKA S, SEGALOFF A AND RUBENS RD. (1977). Assessment of response to therapy in advanced breast cancer. *Cancer*, **39**, 1289-1293.

HEDLEY DW, FRIEDLANDER ML, TAYLOR IW, RUGG C AND MUSGROVE E. (1983). Method for analysis of cellular DNA content of paraffin-embedded pathological materials using flow cytometry. *J. Histochem. Cytochem.*, **31**, 1333-1335.

HEDLEY DW, RUGG CA AND GELBER RD. (1987). Association of DNA index and S-phase fraction with prognosis of node-positive early breast cancer. *Cancer Res.*, **47**, 4729-4735.

JOENSUU H AND TOIKKANEN S. (1992). Identification of subgroups with favorable prognosis in breast cancer. *Acta Oncol.*, **31**, 293-301.

KALLIONIEMI O-P, HIETANEN T, MATTILA J, LEHTINEN M, LAUSLAHTI K AND KOIVULA T. (1987). Aneuploid DNA content and high S-phase fraction of tumor cells are related to poor prognosis in patients with primary breast cancer. *Eur. J. Cancer Clin. Oncol.*, **23**, 277-282.

KALLIONIEMI O-P, BLANCO G AND ALAVIKKO M. *et al.* (1988). Improving the prognostic value of DNA flow cytometry in breast cancer by combining DNA index and S-phase fraction. *Cancer*, **62**, 2183-2190.

MASTERS JRW, CAMPLEJOHN RS, MILLIS RR AND RUBENS RD. (1987). Histological grade, elastosis, DNA ploidy and the response to chemotherapy of breast cancer. *Br. J. Cancer*, **55**, 455-457.

NISKANEN E, FRANSSILA K, BLOMQVIST C, HIETANEN P AND WASENIUS V-M. (1993). The prognostic role of histological grade and c-erbB-2 oncogene amplification in primary tumours of metastatic breast cancer (abstract 427). The Seventh European Conference on Clinical Oncology and Cancer Nursing, Jerusalem, 14-18 November 1993. *Eur. J. Cancer Clin. Oncol.*, **29A** (Suppl. 6), 82.

O'REILLY SM, CAMPLEJOHN RS, RUBENS RD AND RICHARDS MA. (1992). DNA flow cytometry and response to preoperative chemotherapy for primary breast cancer. *Eur. J. Cancer*, **28**, 681-683.

OSBORNE CK. (1989). DNA flow cytometry in early breast cancer: a step in the right direction. *J. Natl Cancer Inst.*, **81**, 1344-1345.

REMVIKOS Y, BEUZEBOC P, ZAJDELA A, VIOLLEMOT N, MAGDALENAT H, POUILLART P. (1989). Correlation of pretreatment proliferative activity of breast cancer with the response to cytotoxic chemotherapy. *J. Natl Cancer Inst.*, **81**, 1383-1387.

REMVIKOS Y, JOUVE M, BEUZEBOC P, VIEHL P, MAGDELENAT H AND POUILLART P. (1993). Cell cycle modifications of breast cancers during neoadjuvant chemotherapy: a flow cytometry study on fine needle aspirates. *Eur. J. Cancer*, **29A**, 1843-1848.

SIGURDSSON H, BALDETORP B, BORG Å, DALBERG M, FERNÖ M, KILLANDER D AND OLSSON H. (1990). Indicators of prognosis in node-negative breast cancer. *N. Engl. J. Med.*, **322**, 1045-1053.

SPYRATOS F, BRIFFORD M, TUBIANA-HULIN M, ANDRIEU C, MAYRAS C, PALLUD C, LASRY S AND ROUESSE J. (1992). Sequential cytopunctures during preoperative chemotherapy for primary breast carcinoma. II. DNA flow cytometry changes during chemotherapy, tumour regression, and short-term follow-up. *Cancer* **69**, 470-475.

STÅL O, WINGREN S, CARSTENSEN J, RUTQVIST LE, SKOOG L, KLINTENBERG C AND NORDENSKJÖLD B. (1989). Prognostic value of DNA ploidy and S-phase fraction in relation to estrogen receptor content and clinicopathological variables in primary breast cancer. *Eur. J. Cancer Clin. Oncol.*, **25**, 301-309.

SULKES A, LIVINGSTON RB, MURPHY WK. (1979). Tritiated thymidine labelling index and response in human breast cancer. *J. Natl Cancer Inst.*, **62**, 513-515.

TOIKKANEN S, JOENSUU H AND KLEMI P. (1989). The prognostic significance of nuclear DNA content in breast cancer - a study with long-term follow-up. *Br. J. Cancer*, **60**, 693-700.

UYTERLINDE AM, BAAK JPA, SCHIPPER NW, PETERSE H, MATZE E AND MEIJER CJL. (1990). Further evaluation of the prognostic value of morphometric and flow-cytometric parameters in breast cancer patients with long follow-up. *Int. J. Cancer*, **45**, 1-7.